# A metasurface carpet cloak for electromagnetic, acoustic and water waves

**DOI:** 10.1038/srep20219

**Published:** 2016-01-29

**Authors:** Yihao Yang, Huaping Wang, Faxin Yu, Zhiwei Xu, Hongsheng Chen

**Affiliations:** 1 State Key Laboratory of Modern Optical Instrumentation, Zhejiang University, Hangzhou 310027, China; 2 Ocean College, Zhejiang University, Hangzhou 310058, China; 3 School of Aeronautics and Astronautics, Zhejiang University, Hangzhou 310027, China

## Abstract

We propose a single low-profile skin metasurface carpet cloak to hide objects with arbitrary shape and size under three different waves, i.e., electromagnetic (EM) waves, acoustic waves and water waves. We first present a metasurface which can control the local reflection phase of these three waves. By taking advantage of this metasurface, we then design a metasurface carpet cloak which provides an additional phase to compensate the phase distortion introduced by a bump, thus restoring the reflection waves as if the incident waves impinge onto a flat mirror. The finite element simulation results demonstrate that an object can be hidden under these three kinds of waves with a single metasurface cloak.

With the development of metamaterial^1^,^2^, it is scientifically possible to realize invisibility cloaks, which have attracted researchers’ great interests in electromagnetic community. Two main strategies, cancellation^3^ and transformation optics^4^,^5^, have been developed to design invisibility cloaks. In the scattering-cancellation based method, a dielectric object is covered by a layer of a plasmonic material. The dipoles in two objects cancel each other out, thus the total scattering will be dramatically reduced. Note the volumetric plasmonic cloak shell can be further replaced with an ultrathin artificial layer, which is the so-called metasurface^6^,^7^. The second method, transformation optics, is proposed by Pendry *et al.*^5^ and Leonhardt^4^. By utilizing the “metric invariance” of Maxwell’s equations, a “hole” in the physical space is created based on a coordinate transformation. Light can be smoothly guided around the hidden object and propagate through the cloak without any perturbation. Because the coordinate transformation is from a point to a line (cylindrical cloak) or a surface (spherical cloak) and the constitutive parameters of the cloaks contain extreme values, an isolated cloak is usually difficult to realize. To get rid of the extreme parameters, carpet cloaks with transformation from a line in virtual space to a different line in physical space were proposed to hide an object on a reflective surface^8^,^9^,^10^. However, the thickness of all the carpet cloaks introduced above is usually comparable with the size of the hidden object. To overcome this challenge, recently, several metasurface carpet cloaks were proposed^11^,^12^,^13^,^14^,^15^,^16^. In a metasurface carpet cloak, the hidden object is covered with an ultrathin gradient metasurface, which is used to control the local reflection phase and restore the reflection waves. These studies show that the metasurface carpet cloak can hide objects with arbitrary shape and size over a wide range of incidence angles and a broad bandwidth^12^ and doesn’t introduce lateral shift as the conventional quasi-conformal mapping based bulk cloak for EM waves^11^,^16^.

As analogs of EM cloaks, the acoustic cloaks also receive increasing interests^17^,^18^, due to their potential applications. With the aid of acoustic metamaterials, some acoustic cloaks have been realized, including scattering cancellation based acoustic cloak^19^, 2D acoustic omnidirectional cloak^20^, 2D acoustic carpet cloak^21^, and 3D acoustic carpet cloak^22^, etc. Note that the concept of invisibility cloak has been also extended to other physical systems, such as linear liquid surface wave^23^, seismic waves^24^, thermal fields^25^,^26^, static magnetic fields^27^ and electric fields^28^, matter waves^29^, etc.

More recently, several attempts have been made to design and realize multiphysics cloaks. Moccia *et al.*^30^ and Li *et al.*^31^ theoretically demonstrated that it is possible to use only one metamaterial structure to independently manipulate both DC current field and heat field. The theory was further validated by Ma *et al.*^32^ The physical mechanism behind this fact is that both thermal conduction equation and electric conduction equation are Laplace equations. Xu *et al.* also found that the cylindrical cloak formerly introduced for linear surface liquid waves works equally well for sound and EM waves^33^. The reason is that the mathematical model of a two-dimensional aluminum cloaking structure (Helmholtz equation with Neumann boundary conditions set at the boundary of the aluminum elements) valid for transverse magnetic (TM) EM waves (aluminum elements behave as perfect electric conductors (PEC) in microwaves). It can also be applied to acoustic waves (they behave like rigid inclusions in air) and water waves (no flow conditions if the aluminum elements is higher than the water surface)^33^.

In this paper, we extend the metasurface carpet cloak concept from EM wave to two other kinds of waves, i.e., acoustics wave and water wave. We propose a single multi-wave metasurface carpet cloak that can manipulate EM wave, acoustic wave, and water wave. Here, ‘multi-wave metasurface’ means a metasurface works for multi waves. The physical mechanism of manipulating three waves with a single cloak is that the same mathematical model of the metasurface cloak can apply for EM TM wave, acoustic wave and water wave. In the following sections, we first introduce the basic principle of our metasurface cloak and then demonstrate the cloaking performance for three kinds of waves. Our simulation results show that a single metasurface carpet cloak can successfully hide the objects under these three waves.

## Theories

**Figure 1:**
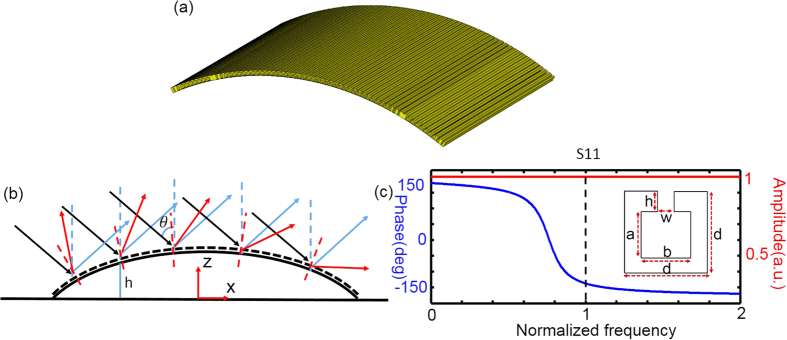
(**a**) Perspective view of a metasurface skin carpet cloak. (**b**) Scheme of a metasurface carpet cloak. The two bold black lines represent the aluminum ground and the bump, respectively. The black dash line is the skin metasurface cloak. The black arrows are the incident waves; the red arrows are the reflected waves without the metasurface cloak; the blue arrows are the reflected waves with the metasurface cloak. The incoming wave is incident with angle of *θ*. *h* is the height of the center point of unit cell from the ground. (**c**) A typical unit cell of the metasurface cloak and its corresponding S11 parameters. The reflection phase of this unit cell can be controlled by altering the geometry of the spilt and cavity. The blue and red lines are the phase and the amplitude of S11 when w = 0.05d, h = 0.3d, a = 0.5d, b = 0.7d, respectively. The dash black line is the operational frequency.

In this section, we introduce the basic idea of a multi-wave metasurface carpet cloak for electromagnetic, acoustic and water waves. As indicated in Fig. 1(b), an incoming wave, with incident angle of *θ*, impinges onto the aluminum bump placed on an aluminum ground, the phase of reflection waves are distorted due to the unnecessary phase introduced by the bump. To compensate for the unwanted phase introduced by the bump and reconstruct the reflection field, an ultrathin skin gradient metasurface is applied to cover the bump, resulting in the recovering of the local reflection phase at each point of metasurface. The local reflection phase is denoted by


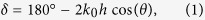


where *h* is the height of the center point of unit cell from the ground, 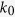 is the wave vector in the surrounding space of the aluminum elements.

The unit cell of metasurface is an aluminum split ring resonator, as shown in the inset of Fig. 1(c). When propagating along –z direction, the waves will go inside the cavity and oscillate in it, and then come back with a phase depending on the geometry of the cavity and the split. In our case, the period of metasurface unit cell is only about 1/8 operational wavelength and the structure is a two-dimensional (2D) structure (or invariant along y direction). In such a two-dimensional space, the boundaries between aluminum elements and air are Neumann boundaries with 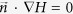 (*H* is the magnetic field) for TM EM wave and 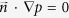 (*p* is the pressure field) for acoustic wave, respectively. The shallow water wave can be treated as a two-dimensional wave, and boundaries between aluminum elements (higher than the water surface) and water are Neumann boundaries with 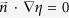 (*η* is the vertical displacement of water wave). Mathematically, the boundaries set at the aluminum structures are the same for these three different physical systems. Besides, the harmonic TM EM, acoustic and water waves are governed by the same equation in a 2D homogeneous space, namely, Helmholtz equation:


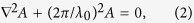


where 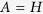, for TM EM wave; 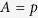, for acoustic wave; 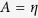, for water wave and 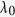 is the wavelength in the homogeneous material. Therefore, the mathematical model of our unit cell in three different physical systems are the same, i.e., Helmholtz equation with Neumann boundary conditions set at the aluminum structures. As *A* in Equ. (2) is only related to the wavelength. Thus if the wavelengths for these three waves are the same, the field distributions will be exactly the same. We show the S11 parameters of a typical unit cell in Fig. 1(c), where w = 0.05*d*, h = 0.3*d*, a = 0.5*d*, b = 0.7*d* (*d* is the period of the unit cell). The horizontal axis is the normalized frequency,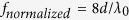, and the vertical axis is the S11 parameters.

## Simulations

In this section, we design two shapes of metasurface cloaks with several different incident angles to demonstrate that our scheme is valid for arbitrary shapes. In the first example, we simulate an arc shape metasurface cloak with the incident angle *θ* as 0°, while in the second example, we model a triangle shaped cloak with the incident angle *θ* as 45 °. The arc curve in our arc shaped cloak is described as:


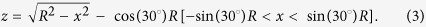


where R is the radius of the arc curve. The local reflection phase, therefore, is


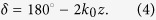


**Figure 2:**
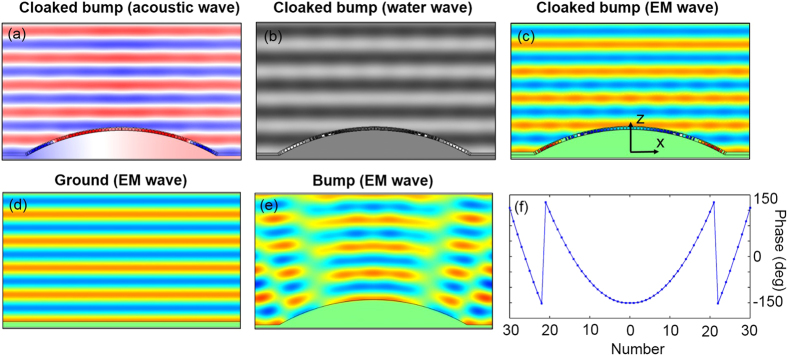
(**a**) Pressure field distributions when an acoustic plane wave is incident onto cloaked bump vertically. (**b**) Vertical displacements of water surface when a plane water wave is incident onto cloaked bump vertically. (**c**–**e**) Magnetic field distributions when an EM plane wave is incident onto cloaked bump, flat ground and bare bump vertically, respectively. (**f**) Local reflection phase of each subwavelength segment.

In our settings, *R* is equal to 57.3*d*, and the metasurface cloak is composed of 60 unit cells. The local reflection phase of each unit cell is shown in Fig. 2(f). The size of each unit cell is shown in Table. S1 in the supplementary information. We model the metasurface cloak by using the commercial finite elements package COMSOL MULTIPHYSICS. In the simulation, d = 7.5 mm, the source is a plane wave propagating along –z direction, perfectly matched layers (PMLs) are applied on all sides of the simulation domain to reduce the scattering and all boundaries are set as scattering boundaries. Figure 2(a–c) show the field distributions near the cloaked bump for acoustic, water and EM TM waves, respectively with the operational wavelength as 60 mm. For brevity, we only show the field pattern when the EM wave illuminates a flat plane and a bare bump, as shown in Fig. 2(d,e). According to the analysis before, the acoustic and water wave cases are the same as that of EM wave. It is obvious that when a plane wave impinges onto the bare bump, the reflected wavefront will be distorted as indicated in Fig. 2(e). Our metasurface cloak can provide an additional phase to compensate for the phase difference introduced by the geometry distortion of the bump, thus strongly suppress the scattering from the bump. The reconstructed phase and amplitude of the reflected wave is almost perfect for these three kinds of waves, i.e., acoustic wave, EM wave, and water wave [see Fig. 2(a–c)].

**Figure 3:**
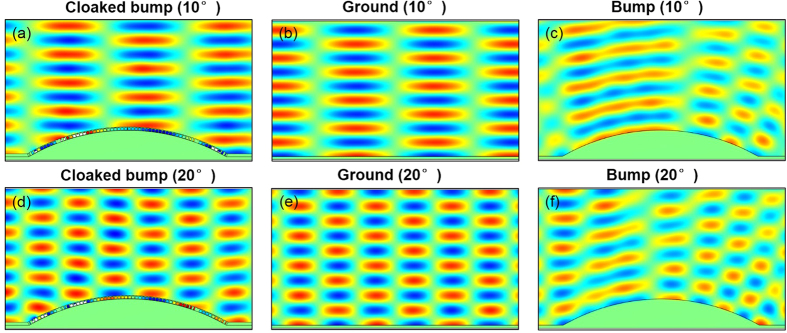
(**a**–**c**) Magnetic field distributions when an incoming EM plane wave illuminate cloaked bump, ground and bare bump, respectively, with 10° incidence angle. (**d**–**f**) Magnetic field distributions when an incoming EM plane wave illuminate cloaked bump, ground and bare bump, respectively, with 20° incidence angle.

Though this metasurface cloak is designed for incident angle of 0°, it can also work on other incident angles. We simulate several different incident angles to demonstrate the operational view angle of our metasurface cloak. When the incidence angle is 10°, the corresponding magnetic field distributions for cloaked bump, ground and bare bump are shown in Fig. 3(a–c), respectively. For simplicity, here we only show the EM wave case. Because the incident wave will overlap with the reflected wave, the wave along z direction is a standing wave as shown in Fig. 3(b). Comparing Fig. 3(a) with (c), it is obvious that the metasurface cloak shows a good cloaking performance. Figure 3(d–f) manifest that the operational view angle can extend to 20°, which means that our cloak can work at least over the range of view angle from −20° to 20°.

In the second example, we design a triangle metasurface carpet cloak for a Gaussian beam with 45° incidence angle. The outline of the triangle bump is expressed as:


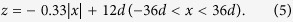


Therefore, the local reflection phase is


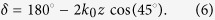


**Figure 4:**
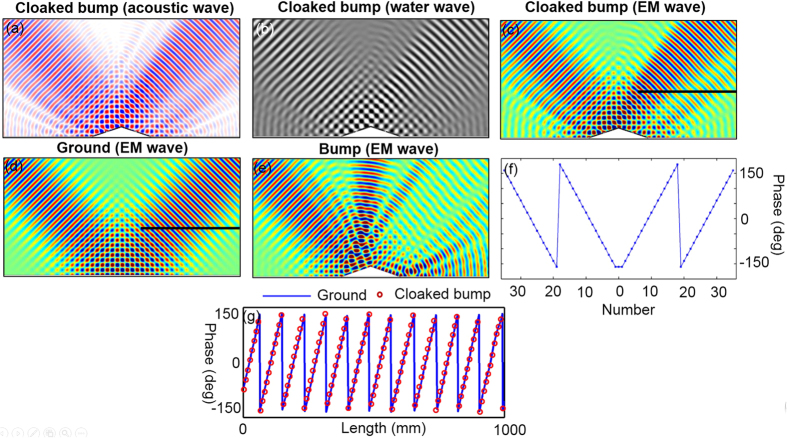
(**a**) Pressure field distributions when an acoustic Gaussian beam is incident onto cloaked bump from top left with incidence angle of 45°. (**b**) Distributions of vertical displacement when a water Gaussian beam is incident onto cloaked bump from top left with incidence angle of 45°. (**c**–**e**) Magnetic field distributions when an EM Gaussian beam is incident onto cloaked bump, ground and bare bump from top left with incidence angle of 45°, respectively. (**f**) Local reflection phase of each subwavelength segment. (**g**) Local phase along the black line marked in (**c**,**d**), where the horizontal axis is the length of the black line from the left endpoint.

**Figure 5:**
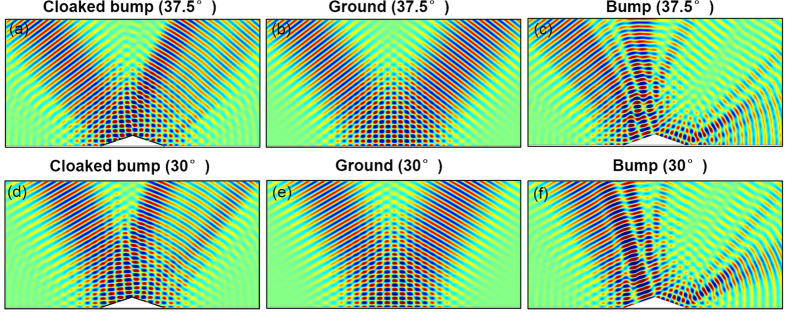
(**a**–**c**) Magnetic field distributions when a Gaussian beam with incident angle as 37.5° impinges onto a cloaked object, flat mirror and bare object, respectively. (**d**–**f**) Magnetic field distributions when a Gaussian beam with incident angle as 30° impinges onto a cloaked object, flat mirror and bare object, respectively.

The local reflection phase of each unit cell is shown in Fig. 4(f). The size of each unit cell is shown in Table. S2 in the supplementary information. In the simulation, the wave source is a Gaussian beam incident from the left top at incident angle of 45°. The field distribution results of three different waves are the same. For simplicity, we only show the EM wave results for bare bump and ground cases. As expected, with only a bare aluminum bump, the reflected wave will be distorted and a large shadow be formed behind the bump, as shown in Fig. 4(e). After covering the bump with a gradient ultrathin metasurface, the distorted reflection waves including EM, acoustics and water waves will be restored and the Gaussian beam profile is reserved as shown in Fig. 4(a–c). For a clearer comparison, we show in Fig. 4(f) the magnetic field phase along the black line marked in Fig. 4(c,d). It is obvious that the field phase line of cloaked bump matches with that of flat mirror very well. Besides, it is interesting to see that our method eliminates the lateral shift which is inevitable for quasi-conformal mapping method^11^,^16^. We also show the field distributions when the incidence angle is 37.5° and 30° in Fig. 5 for EM wave, respectively. The simulation results in Fig. 5 show that this metasurface based cloak works well over a range of incidence angle from 30° to 45°.

## Conclusion

In summary, we proposed a low-profile skin carpet metasurface cloak to hide objects with arbitrary shape and size under electromagnetic, acoustic and water waves. We showed that a single metasurface can control the local reflection phase of these three different waves. By taking advantage of this metasurface, we designed a multi-wave metasurface carpet cloak which can provide an additional phase to compensate for the phase distortion introduced by the bump, thus restoring the reflection waves and reserving the wavefront shape as if the incident waves impinge onto a flat mirror. The finite element simulations demonstrated that we can hide objects under three different waves with just a single metasurface cloak. The tolerated incident angle of the first cloak is at least from −20° to 20° and that of the second cloak is from 30° to 45°. Besides, this metasurface cloak shows no lateral shift of reflection beams.

## Additional Information

**How to cite this article**: Yang, Y. *et al.* A metasurface carpet cloak for electromagnetic, acoustic and water waves. *Sci. Rep.* **6**, 20219; doi: 10.1038/srep20219 (2016).

## Supplementary Material

Supplementary Information
