# Supplementary material for: A metasurface carpet cloak for electromagnetic, acoustic and water waves
Source: Sci Rep. 2016 Jan 29;6:20219. doi: 10.1038/srep20219 (PMC4731745; doi:10.1038/srep20219)
Supplement: Supplementary Information [file srep20219-s1.doc]

*Supplementary Material for*

**A metasurface carpet cloak for electromagnetic, acoustic and water waves**

Yihao Yang^1^, Huaping Wang^2^, Faxin Yu^3^, Zhiwei Xu^2^, Hongsheng Chen^1^

^1^State Key Laboratory of Modern Optical Instrumentation, Zhejiang University, Hangzhou 310027, China.

^2^Ocean College, Zhejiang University, Hangzhou 310058, China.

^3^School of Aeronautics and Astronautics, Zhejiang University, Hangzhou 310027, China

To whom correspondence should be addressed: E-mail: (H. Wang) hpwang@zju.edu.cn and (H. Chen) [hansomchen@zju.edu.cn](mailto:hansomchen@zju.edu.cn).

**Table S1. Geometries of unit cells in the arc shape cloak.**


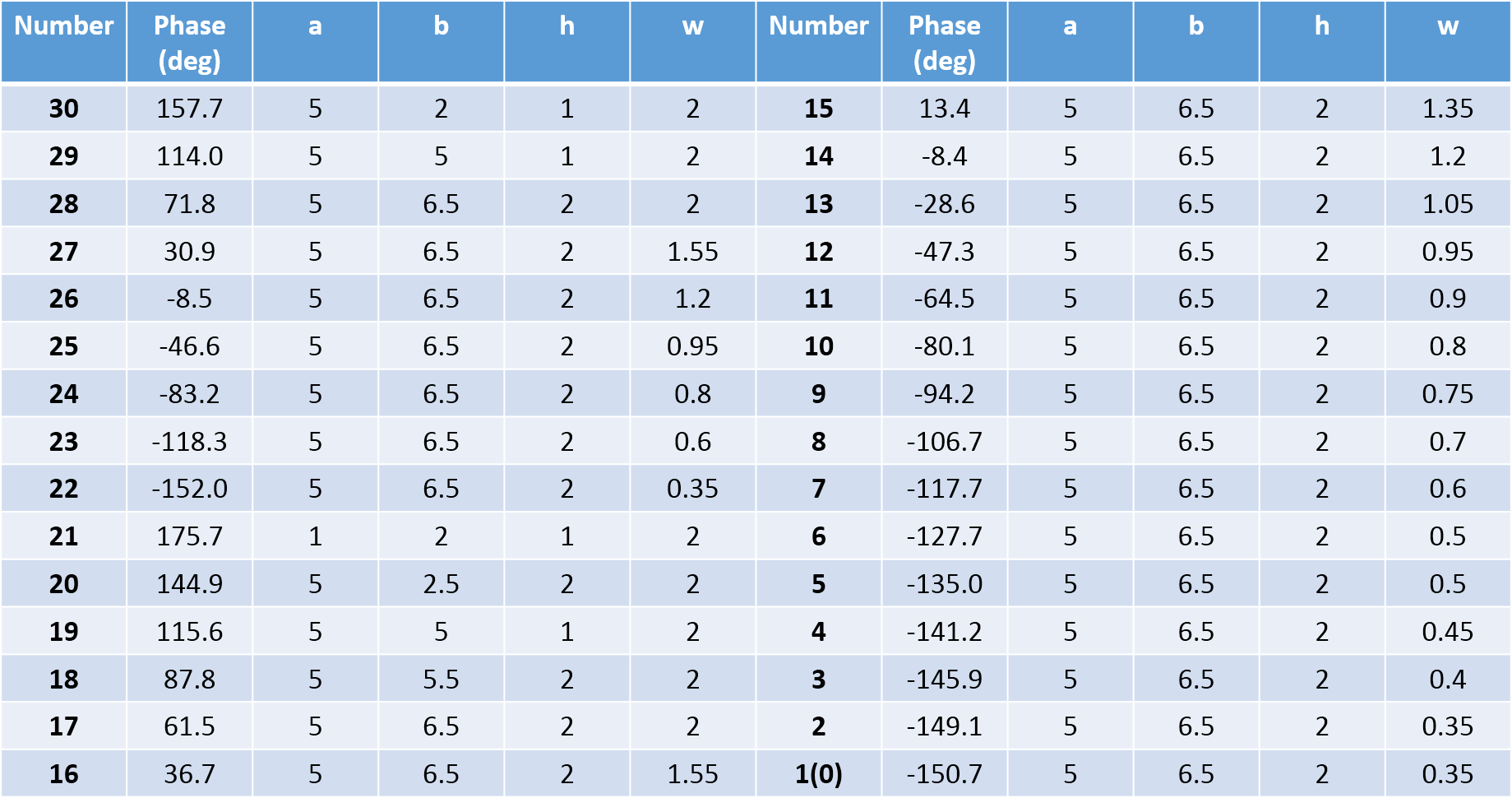


**Table S2. Geometries of unit cells in the triangle shape cloak.**


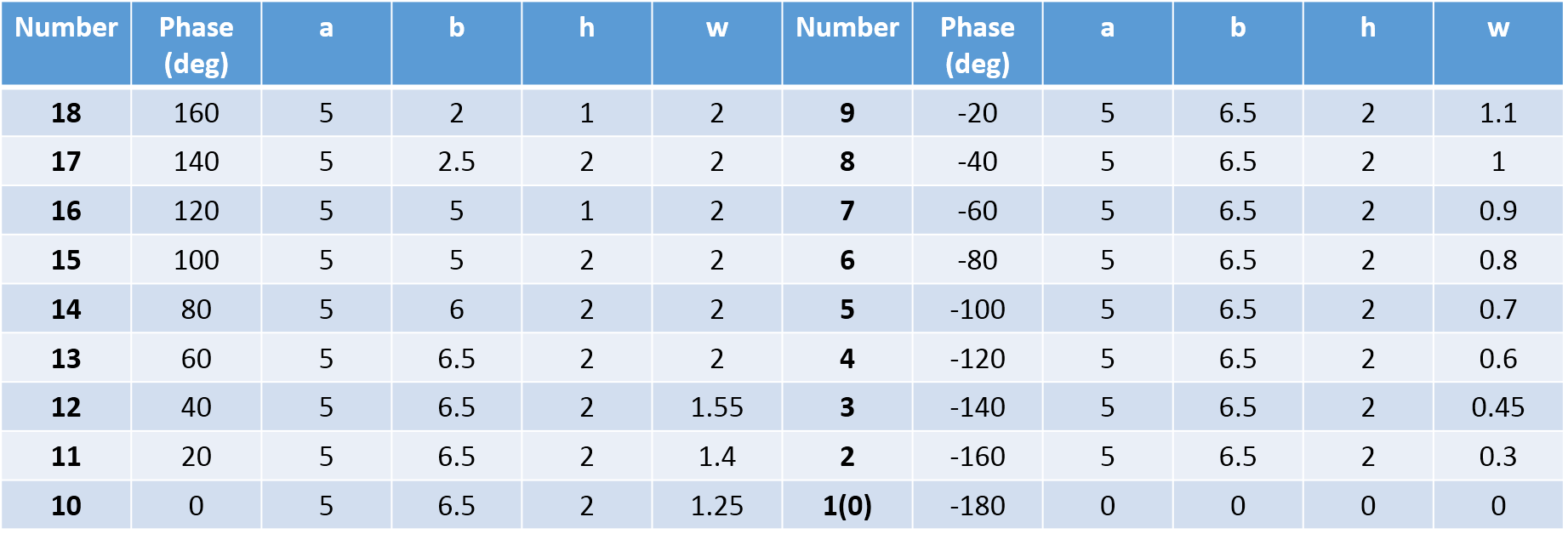


The concrete sizes of unit cell for both arc shape cloak and triangle shape cloak are shown in the Table S1 and Table S2, respectively. We should note that for triangle shape metasurface cloak, the local reflection phases of unit cells from No. 19 to No. 36 are the same as that from No. 1 to No. 18. The unit is millimeter.
